# Behavioral, Psychological, and Metabolic Characteristics of Adults with Co-Occurring Generalized Anxiety Disorder and Obesity: A Cross-Sectional Study

**DOI:** 10.3390/nu18193115

**Published:** 2026-09-22

**Authors:** Natasha Kim de Oliveira da Fonseca, Gabriela Rocha dos Santos, Gabrielle Terezinha Foppa, Gabriella Nunes da Luz Morelo, Artur Zanelatto Santos, Adele Lanziani Faé, Natan Pereira Gosmann, Carolina Blaya Dreher, Luciana Conceição Antunes, Elisa Brietzke, Gisele Gus Manfro

**Affiliations:** 1 Anxiety Disorders Program (PROTAN), Hospital de Clínicas de Porto Alegre (HCPA), Universidade Federal do Rio Grande do Sul (UFRGS), Porto Alegre 90035-903, Brazilgmanfro@gmail.com (G.G.M.); 2 Graduate Program in Psychiatry and Behavioral Sciences, Hospital de Clínicas de Porto Alegre (HCPA), Universidade Federal do Rio Grande do Sul (UFRGS), Porto Alegre 90035-903, Brazil; 3 School of Medicine, Universidade Luterana do Brasil (ULBRA), Canoas 92425-020, Brazil; 4 Laboratory of Neuroscience and Eating Behavior, Universidade Federal de Santa Catarina (UFSC), Florianópolis 88040-900, Brazil; 5 Department of Nutrition, Health Sciences Center, Universidade Federal de Santa Catarina (UFSC), Florianópolis 88040-900, Brazil; 6 Department of Psychiatry, Queen’s University School of Medicine, Kingston, ON K7L 3N6, Canada

**Keywords:** obesity, generalized anxiety disorder, eating behavior, inflammation

## Abstract

**Background:** Generalized Anxiety Disorder (GAD) and obesity are highly prevalent and often co-occur, yet it remains unclear how the presence of both conditions relates to biological, behavioral, and psychological outcomes compared with either condition alone. Understanding this subgroup is crucial to improving assessments and treatment outcomes. **Objective:** To characterize biological, psychological, and behavioral parameters among individuals living with obesity (LWO) and GAD (GAD + LWO), compared with those with only one condition (GAD or LWO) and healthy controls. The primary outcome was to characterize binge eating severity, assessed using the Binge Eating Scale (BES). All remaining behavioral, psychological, and biological outcomes were considered secondary evaluated outcomes. **Methods:** This cross-sectional study included 232 adults: GAD + LWO (*n* = 73), GAD only (*n* = 52), LWO only (*n* = 53), and controls (*n* = 54). Participants completed validated psychological measurements and eating behavior assessments and underwent clinical and metabolic evaluations, including insulin resistance (HOMA-IR) and C-Reactive Protein (CRP). Statistical analyses were performed using analysis of covariance (ANCOVA) for group comparisons and additional interaction analyses between GAD diagnosis and obesity status, adjusted for age and sex. **Results:** Binge eating severity was significantly higher in the GAD + LWO group than in all other groups, with the group mean exceeding the screening threshold for moderate binge eating. This group also showed significantly higher levels of depressive symptoms, emotional eating and impulsivity compared to all other groups. Factorial analyses showed a significant GAD diagnosis × obesity interaction only for depressive symptom severity. Compared to the LWO group, individuals with GAD + LWO also exhibited higher emotional dysregulation, neuroticism, and shame. Biologically, this group showed elevated insulin resistance and higher proportions of systemic inflammation (CRP > 3.0 mg/L) in comparison with the GAD group, but similar to the LWO group. **Conclusions:** The co-occurrence of GAD and LWO was associated with greater severity across several behavioral and psychological outcomes compared with either condition alone. These findings highlight the potential role of tailored, integrated interventions that extend beyond standard obesity treatment to address eating behavior and self-regulation difficulties. Future prospective studies are needed to confirm these exploratory findings and determine their relevance for long-term treatment outcomes and relapse risk.

## 1. Introduction

Obesity is a complex, heterogeneous, and progressive disease, in which abnormal or excess body fat (adiposity) impairs health, increases the risk of long-term medical complications, and reduces lifespan [1]. It is one of the leading global health concerns, contributing to substantial morbidity, mortality, and healthcare costs across diverse populations [2]. Its treatment remains particularly challenging due to its multifactorial nature, high rates of relapse, and the limited long-term effectiveness of most current interventions [3]. Understanding the biological, psychological, and behavioral parameters of chronic diseases such as obesity is essential to improve patient stratification, and develop more effective and personalized treatments, particularly in the context of co-occurring psychiatric disorders.

In the biological context, the co-occurrence of obesity and mental disorders is thought to constitute a specific phenotype with metabolic and neural abnormalities [4,5]. While factors such as peripheral inflammation, hormonal dysregulation, and genetic alterations may play a role, the precise neural circuit mechanisms and key neurotransmitter pathways underlying this co-occurrence remain elusive [5,6,7,8]. In the psychological and behavioral context, emotional and stress-related eating are significant contributors to excessive caloric intake and poor weight outcomes [9]. Anxiety symptoms not only contribute to weight gain and increased appetite but may also stimulate cravings for high-sugar foods [10]. Emotional dysregulation has been linked to eating in response to aversive events among adults living with obesity (LWO) [11]. Negative emotional states can drive dysregulated eating behaviors, such as binge eating, and increase appetitive motivations, including heightened food cravings [12,13]. These behaviors may stem from difficulties in emotional regulation combined with the acute impact of negative affect on self-regulatory processes [14,15]. Despite this, weight loss programs often fail to address the emotional factors underpinning maladaptive eating behaviors [16]. Traditional obesity interventions, such as diet and exercise, may overlook critical behavioral dimensions present in individuals with comorbid psychiatric conditions.

Anxiety disorders are the most prevalent psychiatric conditions, with lifetime prevalence rates ranging from 4.8% to 31% in the general population [17]. Not surprisingly they are also among the most common mental disorders in individuals with obesity [18], particularly Generalized Anxiety Disorder (GAD), Agoraphobia, Panic Disorder, and Post-Traumatic Stress Disorder [19]. Although the literature presents some inconsistencies regarding the co-occurrence of anxiety and obesity, it is well established that this association is more pronounced in females [20,21]. Psychiatric populations are disproportionately affected by physical health conditions, and endocrine and metabolic diseases represent the second leading cause of mortality in these individuals, following unnatural causes such as suicide [22]. This also underscores the urgent need to better understand and manage metabolic risk in individuals with severe psychological disorders. A mental health-informed framework is essential to address these psychological complexities and guide more effective, personalized care.

The relationship of anxiety, obesity and eating behaviors seems to be quite complex and not yet fully investigated. Anxiety disorders have been identified as risk factors for both anorexia [23] and obesity [24]. Evidence suggests that alterations in eating behavior may be influenced by various factors, including personality traits [25], self-control deficits [26], attentional processes [27], and impairments in executive functioning and decision-making [28,29]. Additionally, neuroendocrine and neuropsychological factors play a direct role in the development of obesity, involving disruptions in the reward system, executive control, and autonomic regulation [29].

Although two in every five individuals living with overweight or obesity also meet criteria for a psychiatric disorder [30], treatment remains fragmented across specialties. Patients LWO are usually followed by endocrinologists and dietitians, while those with GAD are treated by psychiatrists. In these separate care settings, behavioral aspects of eating are often neglected. Adherence to lifestyle recommendations is also frequently compromised in psychiatric populations; for instance, a meta-analysis found that only about half of adults with depression adhered to lifestyle interventions [31]. This gap makes it particularly difficult to achieve effective obesity treatment in individuals with GAD, as their specific behavioral patterns are rarely investigated and addressed. Over time, this clinical blind spot may lead to worsening outcomes and increased mortality risk in psychiatric populations LWO.

Given the frequent co-occurrence of anxiety disorder and obesity, and the growing evidence that both conditions independently affect emotional regulation, eating behavior, and treatment adherence, it is essential to investigate how their combination manifests clinically. Does the presence of both conditions result in greater and distinct behavioral and psychological difficulties, or does it primarily reflect the additive burden of two comorbid diagnoses? Clarifying this distinction may improve clinical assessment and support the development of more targeted and effective interventions. To address this question, the present study compared biological, psychological, and behavioral parameters across four groups: individuals living with both GAD and obesity (GAD + LWO), those living with GAD only (GAD), those living with obesity only (LWO), and healthy controls (Control). We hypothesized that individuals with co-occurring GAD and obesity would show higher behavioral and psychological severity, with inflammatory and metabolic alterations paralleling these clinical features.

## 2. Methods

The study was approved by the Research Ethics Committee of Hospital de Clínicas de Porto Alegre—HCPA (CAAE 66476322.5.0000.5327). Recruitment took place from 30 March 2023 to 30 July 2024. All participants signed the written consent form to participate in the present study. All methods were carried out in accordance with relevant guidelines and regulations for research involving human participants.

### 2.1. Participants

The participants of this study are adults aged 18–50 years from the community recruited through media channels, including television, radio, and social media platforms affiliated with the research institution and its investigators. The group GAD + LWO comprised individuals who simultaneously met criteria for both conditions (GAD diagnosis and Body Mass Index [BMI] 30.0–39.9). All participants, regardless of study group, underwent the same standardized psychiatric assessment performed by trained psychiatrists under the supervision of a senior psychiatrist. This assessment included a structured clinical interview using the Mini-International Neuropsychiatric Interview (M.I.N.I.) [32], adapted for DSM-5. The interview was used to confirm eligibility according to group assignment: participants in the GAD and GAD + LWO groups were required to meet DSM-5 criteria for a primary diagnosis of Generalized Anxiety Disorder (participants with other anxiety diagnoses could enter the study provided GAD was the primary diagnosis), whereas participants in the LWO and Control groups were required to have no current DSM-5 psychiatric diagnosis; although binge-eating disorder was not an exclusion criterion and could therefore be present in the LWO-only group. To minimize potential confounding, individuals with a primary diagnosis of Major Depressive Disorder (MDD) or severe depressive symptoms were excluded from all groups. However, participants with comorbid MDD or depressive symptoms were eligible for inclusion provided that MDD was not the primary diagnosis and depressive symptoms were not severe.

The M.I.N.I. is a structured validated clinical diagnostic interview to assess psychiatric diagnoses and is based on the DSM-IV and ICD-10 [32] diagnostic criteria. It has been adapted and validated for use in the Brazilian population [33]. Briefly, two investigators who were clinical psychiatrists and previously trained in the administration of the M.I.N.I. systematically reviewed the interview by comparing each diagnostic module with the corresponding DSM-5 criteria [34]. Specifically, the GAD module was adapted to specify that at least some symptoms associated with worry and anxiety “have to be present for more days than not for the past 6 months”. Binge-eating disorder was assessed during the standardized psychiatric evaluation using a structured interview based on DSM-5 criteria; however, it was not considered an exclusion criterion because of its high comorbidity with obesity and GAD and to preserve the clinical representativeness of the study sample. Therefore, binge-eating symptom severity was assessed dimensionally using the Binge Eating Scale (BES), which was the predefined primary outcome.

The GAD group included participants with a primary diagnosis of GAD and eutrophic weight (BMI between 18.5 and 24.9 kg/m^2^). Individuals with overweight (BMI 25.0–29.9 kg/m^2^) were excluded from all study groups; therefore, only individuals with eutrophic weight or obesity were included. This approach was intended to maximize the contrast between groups, better isolate associations specifically related to obesity, and minimize potential confounding from intermediate metabolic characteristics associated with overweight. The LWO group included individuals with a BMI classified as Grade I or II obesity (BMI between 30.0 and 39.9). Individuals with class III obesity (BMI ≥ 40.0 kg/m^2^) were excluded because of the substantially higher prevalence of metabolic and psychiatric comorbidities in this population, which could increase clinical heterogeneity and confound the interpretation of the findings. The Control group included participants with eutrophic weight (BMI between 18.5 and 24.9) and without psychiatric disorders. Eligibility criteria for each study group are summarized in Table 1.

Exclusion criteria included the presence of any psychiatric disorder in individuals from the LWO-only or Control groups, a BMI below 18.5 in participants from the GAD-only or Control groups, and overweight status (BMI between 25.0 and 29.9) was excluded across all groups. Individuals with GAD were excluded if they had a primary or severe diagnosis of Major Depressive Disorder. Mild-to-moderate depressive symptoms were permitted and were assessed dimensionally using the PHQ-9, reflecting the substantial clinical overlap between anxiety disorders and depressive symptomatology. Furthermore, exclusion criteria also included: (i) current psychopharmacological, psychotherapeutic or structured nutritional treatment at the time of assessment; (ii) a diagnosis of Bipolar Disorder, Schizophrenia-Spectrum Disorder or Substance Use Disorder (except for tobacco use) within the last six months; (iii) Eating Disorders (Anorexia Nervosa or Bulimia Nervosa); (iv) clinical instability or immobility due to medical conditions; (v) pregnancy or lactation, (vi) recent suicidal ideation (during the last six months). The exclusion of individuals with suicidal ideation in the previous six months was adopted for ethical and safety reasons, consistent with standard practices in psychiatric research. The six-month timeframe was chosen as a clinically meaningful period to indicate relative short-term stability of suicidal risk [35].

Participants were also excluded if they reported diabetes mellitus, autoimmune or inflammatory diseases that could influence insulin resistance or inflammatory markers, recent infectious illnesses (participants were rescheduled and assessed only after at least 15 days had elapsed since recovery), or the use of medications known to affect inflammatory markers (e.g., anti-inflammatory or immunomodulatory drugs). Participants with stable chronic conditions (e.g., controlled asthma) were eligible provided they had not experienced an acute exacerbation within the previous month. Medical history and current medication use were systematically assessed during the screening interview for all participants.

### 2.2. Procedures

All individuals were from the community recruited through media advertising. Interested individuals signed up using Google Forms and our team contacted them for screening purposes over the phone. Those who met the inclusion criteria received the informed consent form by email and had a remote psychiatric evaluation scheduled. Four trained physicians interviewed and assessed the participants through structured clinical interviews and other questionnaires to assess GAD diagnosis and symptom severity. All psychiatrist interviewers followed a standardized diagnostic protocol and were trained in the administration of the MINI adapted for DSM-5 criteria. Inter-rater consistency was supported through regular case discussions and supervision by senior psychiatrists, however formal inter-rater reliability was not evaluated. Those who were included were scheduled for the in-person assessment. Two days before the assessment, participants were contacted by the research team and received instructions for blood collection, along with a link to the questionnaires and their unique study ID. Participants were instructed to arrive after at least 8 h of fasting and to complete the self-reported questionnaires before the study visit. A trained professional carried out the assessments: weight, height, abdominal circumference, bioimpedance, clinical, eating and behavioral instruments. Blood samples were collected by venipuncture by a trained phlebotomist for the measurement of glucose, insulin and C-Reactive Protein (CRP). After the assessments, nutritional counseling was provided to all participants.

### 2.3. Measures

#### 2.3.1. Demographic Data

A standardized questionnaire used for the Brazilian population [36] was applied to collect demographic information (age, race/ethnicity, gender and socioeconomic status). Afterwards, the research protocol was implemented with the instruments described below.

#### 2.3.2. Psychological Measures

The Generalized Anxiety Disorder 7-item scale (GAD-7) is a self-administered, brief, validated, and efficient scale for screening and measuring anxiety severity, with excellent internal consistency (Cronbach’s α = 0.92) [37,38]. The Patient Health Questionnaire (PHQ-9) is a brief, self-report tool used to screen for, diagnose, monitor, and measure the severity of depression, with good internal consistency (Cronbach’s α = 0.86) [39,40]. Personality traits were assessed using the Reduced Scale of Big Five Personality Factors (ER5FP), a validated brief instrument designed to assess the five domains of the Big Five personality model (Openness to experience, Conscientiousness, Extraversion, Agreeableness, and Neuroticism), one of the most influential and extensively studied frameworks in personality research; reliability coefficient for the five factors ranged from 0.71 to 0.85 [41,42]. All rating scales are validated to the Brazilian–Portuguese population with good psychometric properties. The Other as Shamer Scale (OAS-2) is a scale that assesses shame, more specifically, the overall judgment of how the subject believes other people think they are perceived. This instrument showed good internal consistency (Cronbach’s α = 0.82) and was administered using the validated European Portuguese version [43,44].

#### 2.3.3. Behavioural Measures

The Difficulties in Emotion Regulation Scale (DERS) is an instrument that assesses levels of emotional dysregulation in six domains, has demonstrated high internal consistency (Cronbach’s α = 0.93), and was administered using the validated European Portuguese version [45,46]. The Barratt Impulsiveness Scale (BIS-11) is a self-report scale developed by Barratt to assess these dimensions of impulsivity and consists of 20 questions for Inhibitory Control and 8 for Lack of Planning; the internal consistency was acceptable (Cronbach’s α = 0.79). The validated Brazilian Portuguese version was administered in the present study [47,48].

#### 2.3.4. Eating Behavior Measures

The Binge Eating Scale (BES) is a self-administered Likert questionnaire consisting of a list of 16 items, from which the one that best represents the individual’s response must be selected for each item [49,50]. BES scores were interpreted according to established screening cut-offs: scores ≤ 17 indicate absence of binge eating, scores of 18–26 suggest moderate binge eating, and scores ≥ 27 indicate severe binge eating. Binge eating behavior was assessed dimensionally and was not used as an inclusion or exclusion criterion in order to preserve external validity. The internal consistency of this instrument was moderately high (Cronbach’s α = 0.83) and was administered using the validated Brazilian Portuguese version. The Three Factor Eating Questionnaire (TFEQ-R21) is a self-administered instrument that assesses behaviors of cognitive restraint (six questions), emotional eating (six questions), and lack-of-control overeating (nine questions) and has good internal consistency. The validated Brazilian Portuguese version was administered in the present study [51,52]. The Power of Food Scale (PFS) is a self-administered scale designed to measure the hedonic impact in environments with abundant food, occurring even in the absence of physical hunger. The validated European Portuguese version was administered in the present study and has demonstrated excellent internal consistency (Cronbach’s α = 0.93) [53,54]. The food consumption questionnaire from the Surveillance of Risk and Protective Factors for Chronic Diseases by Telephone Survey (VIGITEL), an epidemiological surveillance instrument, included two modules: one assessing usual food consumption and another module with questions about consumption on the previous day (24 h) including a subgroup of ultra-processed food (UPF-VIGITEL). These items were selected based on foods most frequently consumed in Brazil according to the national food consumption survey conducted as part of the Consumer Expenditure Surveys (POF) by the Brazilian Institute of Geography and Statistics (IBGE) [55].

#### 2.3.5. Biological Measures

Weight was measured on a calibrated digital scale and height with a vertical stadiometer to determine BMI. Body fat percentage was measured through segmental multi-frequency direct bioelectrical impedance analysis, using the InBody 370s (InBody Co., Ltd., Cheonan, Republic of Korea). Waist circumference was measured at the level of the umbilical scar using standardized anthropometric procedures, with measurements obtained in duplicate using an inelastic measuring tape (Cescorf Equipamentos, Porto Alegre, Brazil). Systemic inflammation was assessed using CRP, with values > 3.0 mg/L considered elevated [56].

Glucose, insulin, and CRP concentrations were assessed immediately upon collection by the certified clinical laboratory of Hospital de Clínicas de Porto Alegre, ensuring standardized and validated methodology. Glucose (mg/dL) was measured using an enzymatic hexokinase/G-6-PDH assay and CRP (mg/L) by immunoturbidimetry on the Abbott Alinity C platform (Abbott Laboratories, Abbott Park, IL, USA). Insulin (µIU/mL) was measured using a chemiluminescent microparticle immunoassay (CMIA) on the Abbott Alinity I platform (Abbott Laboratories, Abbott Park, IL, USA). The intra- and inter-assay coefficients of variation were 0.88% and 1.31% for glucose at control level 1 and 0.72% and 1.13% at control level 2; 3.21% and 3.66% for insulin at control level 1 and 1.73% and 3.09% at control level 2; and 2.83% and 2.90% for CRP at control level 1 and 1.56% and 1.79% at control level 2, respectively. We calculated insulin resistance with an easy to apply method using the HOMA index (fasting glucose [mg/dL] × fasting insulin [µIU/mL]/405).

Biological measurements were collected under standardized conditions following institutional protocols. Blood samples were obtained in the morning after an overnight fast of at least 8 h. Body composition was assessed using bioimpedance analysis according to standardized preparation guidelines, including a minimum fasting period of 4 h for food and fluids, avoidance of intense physical activity and alcohol consumption in the 24 h preceding the assessment, removal of metallic objects, bladder emptying prior to the examination, and postponement of assessment in the presence of fever or pregnancy. All bioimpedance measurements were performed using the same device by trained personnel, following manufacturer and institutional recommendations. Measurements were repeated when technical errors or implausible values were identified.

### 2.4. Data Analysis

This is an exploratory cross-sectional study. Descriptive statistics are presented in the text and tables, including means and standard deviations (SD) and were compared using one-way analysis of variance (ANOVA), medians and interquartile ranges (IQR), or absolute and relative frequencies (%), as appropriate. The normality of continuous variables was assessed using the Shapiro–Wilk test, along with visual inspection of histograms and Q–Q plots.

The analytical strategy followed a hierarchical approach, progressing from group-level comparisons to domain-specific explorations. Group differences were examined using analysis of covariance (ANCOVA), with the GAD + LWO group serving as the case group (reference) compared to the other three (GAD-only, LWO-only, and Control). Post hoc comparisons were conducted using estimated marginal means with Bonferroni-adjusted *p*-values. Cohen’s d effect sizes and their corresponding 95% confidence intervals were calculated for the adjusted post hoc comparisons using the Jamovi ANCOVA procedure; confidence intervals were not adjusted for multiplicity. Effect sizes were interpreted according to Cohen’s conventions (small: 0.2, medium: 0.5, large: 0.8). To account for multiple outcome comparisons, *p*-values were adjusted using the Benjamini–Hochberg step-up false discovery rate (FDR) procedure implemented in WINPEPI version 11.65. To formally evaluate whether GAD diagnosis and obesity status interacted in relation to the study outcomes, we performed 2 × 2 factorial ANCOVA models including GAD diagnosis (present vs. absent), obesity status (present vs. absent), and their interaction, adjusted for age and sex using SPSS v18.0.

Because HOMA-IR and CRP showed positively skewed distributions, both variables were log10-transformed before the analyses to better meet the assumptions of the parametric models. All statistical analyses were performed using the transformed variables. For ease of clinical interpretation, the corresponding raw laboratory values in the original measurement scale are presented in brackets in the tables.

Age and sex were specified a priori as covariates in the primary models because of their well-established associations with GAD, obesity, and several of the behavioral, psychological, and metabolic outcomes examined. Given the relatively modest sample size and the number of outcomes assessed, a parsimonious adjustment strategy was adopted to preserve model precision. Although race/ethnicity has also been associated with GAD, obesity, and related health outcomes in the previous literature, its role as a necessary confounder was considered less consistently supported a priori than that of age and sex. Race/ethnicity was therefore evaluated in sensitivity analyses rather than included in the primary models. The primary estimand was the adjusted difference in each outcome between the GAD + LWO group and each comparison group, conditional on age and sex. Socioeconomic status did not differ significantly across groups and was therefore not included in the main models to preserve parsimony and statistical power. Sex × group interaction analyses were conducted as sensitivity analyses, given the limited number of male participants in some groups. Model assumptions were evaluated prior to analysis. The normality of residuals was assessed through visual inspection of Q–Q plots, and the homogeneity of regression slopes was examined by testing interactions between covariates and group. No violations of ANCOVA assumptions were identified.

A total sample size of 192 participants (48 per group) was calculated for a one-way ANOVA with four groups (controls, GAD, LWO, GAD + LWO). Expected differences in Binge Eating Scale (BES) means assumed as 13.4, 14.5, 16.5, and 18.5 units, were informed by prior clinical literature and large population studies on binge eating behavior in adults with overweight or obesity, including Almeida et al. [57], with a common SD of 8, α = 0.05, and 80% power. Allowing for 10% attrition, the planned sample size was 216 participants. The calculation was performed using the PSS Health (Power and Sample Size for Health Researchers) v0.5.1 online tool [58].

Group differences in the proportion of individuals with elevated CRP (>3.0 mg/L) were analyzed using chi-square tests, with pairwise comparisons restricted to the GAD + LWO group versus each of the other study groups. No multiplicity adjustment was applied to these pairwise comparisons. To provide interpretable effect estimates, binomial logistic regression was additionally performed using elevated CRP as the dependent variable and study group as the predictor, with GAD + LWO as the reference group; odds ratios (ORs) with 95% confidence intervals were reported.

Missing data were handled using complete case analysis, with participants excluded only from analyses involving variables with missing values. The data were analyzed with the statistical software Jamovi v2.5.8 and SPSS v18.0. The tests were two-tailed, with a significance level of 0.05.

## 3. Results

A total of 877 individuals were screened in the study (Figure 1), but data collection was completed for 232 participants, distributed as follows: GAD + LWO (*n* = 73), GAD (*n* = 52), LWO (*n* = 53), and Control (*n* = 54), exceeding the planned size and thereby preserving the intended statistical power. Demographic data are presented in Table 2. The mean age was 34 years (±8), and 194 participants (83.6%) were female. ABEP socioeconomic classification showed that the majority of individuals were in classes B (upper-middle income) and C (lower-middle income). A total of 166 participants (73.8%) self-identified as White. The “Other” category included participants who identified as Asian (*n* = 1) or selected another race/ethnicity (*n* = 5), due to small subgroup sizes.

Among participants with GAD, psychiatric comorbidities permitted by the study inclusion criteria were common in both groups. However, participants with Major Depressive Disorder could enter the study provided it was not the primary diagnosis, nor with severe symptomatology. In the GAD + LWO group, 38.4% (28/73) had a current major depressive episode, 16.4% (12/73) persistent depressive disorder, 19.2% (14/73) agoraphobia, 11.0% (8/73) social anxiety disorder, and 50.7% (37/73) binge-eating disorder. Corresponding proportions in the GAD-only group were 23.5% (12/51), 5.9% (3/51), 7.8% (4/51), 9.8% (5/51), and 19.6% (10/51), respectively. Diagnostic data were unavailable for one participant in the GAD-only group. Among LWO participants with available diagnostic data for BED, 11.3% (6/53) met criteria for current BED. By study design, participants in the LWO-only and control groups did not meet criteria for the psychiatric diagnoses specified as exclusionary for these groups, although binge-eating disorder was not an exclusion criterion and could therefore be present in the LWO-only group.

Table 3 presents group differences using the GAD + LWO group as the reference category in comparisons against the other three groups. Additionally, post hoc pairwise comparisons among the remaining groups are reported in the final column of the table. Based on the a priori sample size calculation, BES was designated as the primary outcome. All remaining behavioral, psychological, and biological measures were evaluated as secondary outcomes.

To formally evaluate whether GAD diagnosis and obesity status interacted in relation to the study outcomes, we performed 2 × 2 factorial ANCOVA models including GAD diagnosis (present vs. absent), obesity status (present vs. absent), and their interaction, adjusted for age and sex. A significant GAD diagnosis × obesity interaction was observed only for depressive symptom severity (PHQ-9; B = 2.889, SE = 1.307, 95% CI 0.310–5.468, t = 2.210, *p* = 0.028, partial η^2^ = 0.026). The interaction for binge eating (BES) was not statistically significant (B = 2.896, SE = 1.950, 95% CI −0.947–6.739, t = 1.485, *p* = 0.139, partial η^2^ = 0.010), and no significant GAD diagnosis × obesity interactions were observed for the remaining outcomes.

To address the marked sex imbalance, sensitivity analyses including sex × group interaction terms were conducted for all outcomes and did not indicate that sex altered the pattern of group differences, supporting the robustness of the main findings (Table S1). Sensitivity analyses additionally adjusting for race/ethnicity yielded largely consistent results (Table S2), with only minor variations in specific outcomes, indicating that the overall pattern of group differences was preserved.

### 3.1. Differential Characteristics in Patients with GAD + LWO

#### 3.1.1. Behavioral Outcomes

Across behavioral domains, the GAD + LWO group consistently showed greater severity than all other groups (Figure 2). Binge eating (BES) in this group was also markedly higher compared to the LWO group (difference = 7.1, *p* < 0.001), the GAD group (difference = 10.1, *p* < 0.001), and the control group (difference = 14.3, *p* < 0.001). Notably, only the GAD + LWO group had a mean BES score within the moderate binge eating severity range. When participants were classified according to established BES severity categories, the distribution was as follows: in the GAD + LWO group, 16 (26.7%) participants were classified as having absence of binge eating, 22 (36.7%) as moderate, and 22 (36.7%) as severe; in the LWO group, 26 (60.5%), 13 (30.2%), and 4 (9.3%), respectively; in the GAD group, 29 (67.4%), 13 (30.2%), and 1 (2.3%); and in the control group, 42 (87.5%), 5 (10.4%), and 1 (2.1%). Difficulties in emotion regulation (DERS) in GAD + LWO were also pronounced, with higher scores than the LWO group (difference = 17.3, *p* = 0.006) and the control group (difference = 22.1, *p* < 0.001).

Impulsivity (BIS-11) followed a marked elevation in the GAD + LWO group compared with all groups, with significantly elevated scores relative to the LWO group (difference = 10.5, *p* < 0.001), the GAD group (difference = 7.2, *p* = 0.004) and controls (difference = 12.1, *p* < 0.001). Emotional eating (TFEQ-EE) was substantially greater in the GAD + LWO group compared with all groups, significantly elevated scores relative to the LWO group (difference = 2.4, *p* = 0.022), the GAD group (difference = 4.5, *p* < 0.001) and controls (difference = 5.3, *p* < 0.001). Uncontrolled eating (TFEQ-UE) in GAD + LWO were also pronounced, with higher scores than the GAD group (difference = 5.0, *p* < 0.001) and controls (difference = 5.7, *p* < 0.001). In contrast, cognitive restraint (TFEQ-CR) did not differ significantly across groups.

Elevated hedonic hunger (PFS) distinguished the GAD + LWO group from both the GAD group (difference = 0.65, *p* < 0.001) and controls (difference = 0.71, *p* < 0.001), while no statistically significant difference was observed compared to the LWO group (difference = 0.42, *p* = 0.059). In turn, consumption of ultra-processed foods (VIGITEL-UPF) did not differ significantly across groups. In sensitivity analyses additionally adjusted for race/ethnicity, most associations remained materially unchanged (Table S2); however, impulsivity in the GAD group was attenuated (*p* = 0.080), while hedonic hunger in the LWO group reached statistical significance (*p* = 0.040).

#### 3.1.2. Psychological Outcomes

The GAD + LWO group also exhibited a significantly higher psychological burden across multiple measures as compared with the other groups. Depressive symptom severity (PHQ-9) was higher than in the LWO (difference = 7.2, *p* < 0.001), GAD (difference = 4.7, *p* < 0.001), and control (difference = 9.0, *p* < 0.001) groups (Figure 2). Elevated neuroticism (ER5FP) distinguished this group from both LWO (difference = 1.2, *p* < 0.001) and controls (difference = 1.3, *p* < 0.001). Shame (OAS-2) was also increased compared to LWO (difference = 4.7, *p* < 0.001) and controls (difference = 6.1, *p* < 0.001). Despite these pronounced differences, anxiety symptom severity (GAD-7) did not differ significantly between the GAD + LWO and GAD groups.

#### 3.1.3. Biological Outcomes

Biological markers paralleled the behavioral and psychological findings. The GAD + LWO group demonstrated significantly higher insulin resistance (HOMA-IR_log) compared to the GAD group (difference = 0.3, *p* < 0.001) and the control group (difference = 0.3, *p* < 0.001), however did not differ significantly from the LWO group. Similarly, systemic inflammation (CRP-log) was greater than in the GAD (difference = 0.3, *p* < 0.001) and control (difference = 0.4, *p* < 0.001) groups, with no difference observed relative to LWO. Figure 3 illustrates the differences in the GAD + LWO group across all comparisons and analyses.

### 3.2. Inflammation Based on CRP Levels

Significant differences were observed in the proportion of individuals with elevated CRP levels (>3.0 mg/L) across groups (χ^2^(3) = 31.2, *p* < 0.001; Cramér’s V = 0.386). More than 60% of individuals in the GAD + LWO (62.7%) and LWO (60.4%) groups had elevated CRP, compared to only 26.7% in the GAD group and 20.4% in controls. To minimize the potential influence of acute inflammatory conditions, a sensitivity analysis excluding participants with CRP values >10 mg/L was performed. The results remained materially unchanged, with a significant association between study group and CRP category (χ^2^(3) = 26.6, *p* < 0.001; Cramér’s V = 0.368). Pairwise comparisons revealed that the GAD + LWO group had a significantly higher proportion of individuals with CRP elevation than both the GAD-only and control groups (*p* < 0.01), but not in comparison to the LWO group.

In complementary binomial logistic regression analyses using GAD + LWO as the reference group, the odds of elevated CRP were lower in Controls (OR = 0.16, 95% CI 0.07–0.39, *p* < 0.001) and in the GAD group (OR = 0.23, 95% CI 0.10–0.54, *p* < 0.001), whereas the LWO group did not differ significantly from GAD + LWO (OR = 0.88, 95% CI 0.40–1.93, *p* = 0.745).

## 4. Discussion

In individuals living with GAD and obesity (GAD + LWO), we observed not only the presence of both conditions, but also greater depressive symptoms, as well as a more severe presentation of emotional eating, impulsivity, and binge eating. Notably, the factorial analysis showed a significant GAD × obesity interaction only for depressive symptom severity. Binge eating severity in this group reached the moderate range, while all other groups remained below this threshold according to the BES.

Within this framework, binge eating may reflect processes related to emotional dysregulation and avoidance-based coping in individuals with GAD, favoring food choices driven by immediate palatability. In this context, threat-focused cognitive tendencies associated with GAD, such as hypervigilance and excessive worry, may impair top-down regulatory processes, limiting flexibility in food-related decisions and facilitating loss of control overeating [59]. Beyond its behavioral manifestation, binge eating may also function as a clinically meaningful indicator of illness severity. Binge eating is associated with greater psychological distress, functional impairment, and symptom burden, while simultaneously being reinforced by these same factors through a bidirectional relationship [60]. Accordingly, interventions targeting affective regulation and depressive symptomatology may represent clinically relevant therapeutic pathways.

The GAD + LWO group showed greater severity across several behavioral and psychological outcomes, including binge eating, emotional eating, impulsivity, and emotion dysregulation. These between-group differences reflect greater symptom severity across the study groups, whereas the factorial analyses specifically tested whether GAD and obesity interacted in relation to these outcomes. A statistically significant GAD × obesity interaction was observed only for depressive symptom severity, indicating that the association with depressive symptoms differed according to the joint presence of GAD and obesity. However, because the study was cross-sectional and was not specifically powered to detect factorial interaction effects, this finding should be interpreted cautiously and confirmed in larger longitudinal studies.

Beyond the clinical manifestation of GAD, individuals with both conditions (GAD + LWO) also exhibited a pattern of psychological and cognitive characteristics that were not observed in the LWO group alone, including elevated neuroticism and shame, greater impulsivity, and more pronounced difficulties in emotion regulation. These characteristics may have implications for treatment adherence and response to conventional obesity treatment; however, these hypotheses require further investigation in prospective studies. They may also increase vulnerability to internalized weight stigma, a construct that has recently been associated with anxiety, shame, emotion dysregulation, and body appreciation in adults with obesity and GAD [61]. This pattern reinforces the importance of considering tailored, mental health–informed eating behavioral interventions for this subgroup, rather than applying a one-size-fits-all model [62]. Furthermore, future research should explore whether early optimization of psychiatric or obesity pharmacotherapy enhances adherence and clinical outcomes in individuals with co-occurring anxiety and obesity.

While metabolic and dietary mechanisms have been the primary focus of obesity research [63], future studies should examine whether incorporating psychological and behavioral profiling could enhance the personalization and effectiveness of nutritional interventions [64]. Given their elevated neuroticism, impulsivity, and greater difficulty with emotion regulation, these individuals may struggle to flexibly manage small portions of hyperpalatable foods, particularly in the context of heightened psychiatric symptoms. Previous literature suggests that individuals with co-occurring GAD and LWO may face additional challenges when engaging in lifestyle-based interventions. Consistent with cognitive-behavioral theory and prior evidence [65], addressing clinically significant psychiatric symptoms may facilitate engagement with structured dietary and behavioral interventions in some individuals.

In parallel, the GAD + LWO exhibited higher inflammatory marker levels and HOMA-IR than the GAD group. However, these differences appeared to be primarily attributable to obesity, indicating that these alterations are largely attributable to excess adiposity rather than anxiety itself, as no significant differences were observed between the GAD + LWO and LWO groups. Likewise, the GAD-only group did not differ significantly from controls regarding the proportion of individuals with CRP levels above 3.0 mg/L. These results are consistent with findings from Copeland et al. (2012), who reported that associations between GAD and CRP were attenuated after adjustment for health-related factors, particularly BMI [66]. The association between GAD and systemic inflammation may be more evident in the context of metabolic dysregulation, whereas depression has been shown to have a direct and independent association with CRP levels [66].

This interpretation is further supported by the lack of an independent inflammatory signal in the GAD-only group, despite the use of conventional rather than high-sensitivity CRP assays in our analyses. Conventional CRP is less sensitive to low-grade systemic inflammation than high-sensitivity CRP assays, which may limit the detection of subtle inflammatory differences. In addition, the use of a 3.0 mg/L threshold, despite it being used in the current literature [56], may result in misclassification of individuals with values near this cutoff, particularly in the context of chronic low-grade inflammation. Therefore, inflammatory findings should be interpreted cautiously, especially when comparing groups with similar levels of adiposity.

Although the metabolic alterations observed in the GAD + LWO group appear to be primarily driven by obesity, these biological processes may interact bidirectionally with mental health. Metabolic dysregulation has been associated with changes in cognitive function, mood regulation, and inflammatory signaling, which may exacerbate psychological factors [67]. Conversely, anxiety-related symptoms and behavioral patterns may influence lifestyle choices and stress-related pathways that negatively affect metabolic regulation [67]. This reciprocal interaction may contribute to the greater clinical complexity observed in individuals with co-occurring GAD and obesity.

One limitation of our study is the use of conventional CRP rather than high-sensitivity CRP (hs-CRP). The 3.0 mg/L threshold applied in our analysis is based on guidelines from the American Heart Association and CDC for hs-CRP assays, which are designed to detect low-grade inflammation more precisely. Consequently, our estimates of systemic inflammation may be less sensitive and should be interpreted with caution, particularly in the context of chronic low-grade inflammatory states such as those associated with obesity and anxiety. Nonetheless, the use of conventional CRP reflects the resources currently available within the Brazilian public health system, alignment with real-world clinical settings.

The cross-sectional and exploratory design of the study limits to establish causal inferences. Additionally, sample limitations including its size and convenience may have limited the generalizability of the findings. Because participants were recruited through media advertisements, self-selection bias cannot be excluded. Furthermore, the exclusion of individuals receiving ongoing psychiatric or obesity treatment may limit the generalizability of the findings to treatment-naïve populations. Several outcomes were assessed using self-report questionnaires, which may be subject to reporting bias. Although all diagnostic interviews were conducted by trained evaluators under specialist supervision, formal inter-rater reliability was not assessed. Although the overall pattern of findings remained unchanged after FDR adjustment, the secondary outcomes should be interpreted cautiously given the number of outcomes evaluated and require confirmation in future studies.

Although limited by its cross-sectional design and convenience sampling, the observed associations across behavioral, psychological, and biological parameters highlight relevant patterns that warrant further investigation. Future research should adopt longitudinal designs to clarify temporal and causal relationships, integrate behavioral and biological data to test multimodal intervention models, and explore inflammatory and metabolic biomarkers as potential mediators of treatment response. In addition, unmeasured factors such as physical activity levels, stress, sleep patterns, smoking, hormonal therapies, and other medical conditions may have influenced the observed associations and should be considered as potential confounders in future studies.

The predominance of women in the present sample is consistent with epidemiological data indicating a higher prevalence of anxiety disorders among women and greater female participation in mental health research. Moreover, recruitment strategies emphasizing anxiety, eating behavior, and obesity may have further increased female participation, as women tend to report higher concern and help-seeking related to emotional and behavioral aspects of eating and weight. Generalizability is also limited by the sample’s sociodemographic profile, which was predominantly female, White, and highly educated, a pattern commonly observed in mental health research and digital intervention studies [68,69]. Similar recruitment profiles have been reported in recent Brazilian studies, including digital psychiatry trials, which consistently show higher participation among women with higher educational levels [70].

To address the marked sex imbalance, sensitivity analyses including sex × group interaction terms were conducted for all outcomes and did not indicate that sex altered the pattern of group differences. Minor differences were observed for hedonic hunger and ultra-processed food intake, suggesting that these sensitivity analyses should be interpreted as supportive rather than confirmatory. In addition, as the study was not intended to estimate population-level prevalence, no weighting or post hoc adjustments were applied. Consequently, the results reflect associations within a female-dominant sample and should be generalized with caution.

This study’s strength lies in an extensive psychiatric diagnosis, its comprehensive evaluation, integrating eating behavior patterns, psychological measures, personality traits, as well as metabolic and inflammatory markers, providing a multidimensional perspective on the complex interplay between anxiety and obesity. Moreover, this is the first study focusing specifically on this clinical overlap, a frequent, difficult-to-treat [31], and understudied condition, highlighting the need for more targeted research and personalized treatment approaches.

Taken together, the findings underscore the importance of shifting the focus beyond *what* individuals are eating to also consider *how* and *why* they are eating. Understanding the underlying emotional and psychological drivers of eating behavior may provide more effective strategies for managing obesity, especially when psychiatric conditions are present.

Within this context, our findings suggest that individuals living with GAD and obesity may represent a subgroup with greater clinical complexity. The combination of elevated binge eating, emotional distress, inflammatory markers, and behavioral traits such as impulsivity and shame suggests a pattern of clinical features that may impact treatment adherence and outcomes. Given the exploratory and cross-sectional nature of the study, they reinforce the need to consider comorbid psychiatric symptoms when assessing and treating individuals with obesity. Future studies are needed to confirm whether this subgroup may benefit from more personalized, psychologically informed interventions alongside conventional obesity care.

## 5. Conclusions

In individuals living with GAD and obesity (GAD + LWO), we observed greater severity across several behavioral and psychological outcomes compared with the other study groups, including higher levels of depressive symptoms, binge eating, emotional eating, impulsivity, and emotion dysregulation. However, factorial analyses showed a significant GAD diagnosis × obesity interaction only for depressive symptom severity, indicating that the broader differences observed across the other outcomes should be interpreted as between-group differences rather than as evidence of an interaction between the two conditions. Notably, mean BES scores in the GAD + LWO group fell within the moderate severity range, whereas mean scores in all other groups remained below this threshold.

Compared to individuals in the GAD group, the GAD + LWO group also exhibited higher levels of metabolic and inflammatory markers, including insulin resistance and elevated C-reactive protein. The GAD + LWO group demonstrated greater severity of depressive symptoms and binge eating, as well as higher levels of impulsivity, emotional eating, uncontrolled eating, and hedonic hunger than the GAD group alone.

Furthermore, when compared specifically to the LWO group alone, individuals with both conditions demonstrated additional personality (e.g., neuroticism), behavioral (e.g., impulsivity, emotional and uncontrolled eating) and psychological characteristics (e.g., shame and difficulties in emotion regulation) that may negatively impact engagement and response to obesity treatment if left unaddressed. These observations also highlight the potential value of routine mental health screening in obesity care, particularly for symptoms of anxiety, depression, and binge eating. Together, these findings highlight the importance of considering psychological and behavioral characteristics when evaluating individuals with co-occurring GAD and obesity. Future studies should investigate whether these characteristics have implications for personalized treatment approaches, combining conventional lifestyle interventions with strategies focused on emotional regulation, impulsivity, and eating behavior patterns.

## Figures and Tables

**Figure 1:**
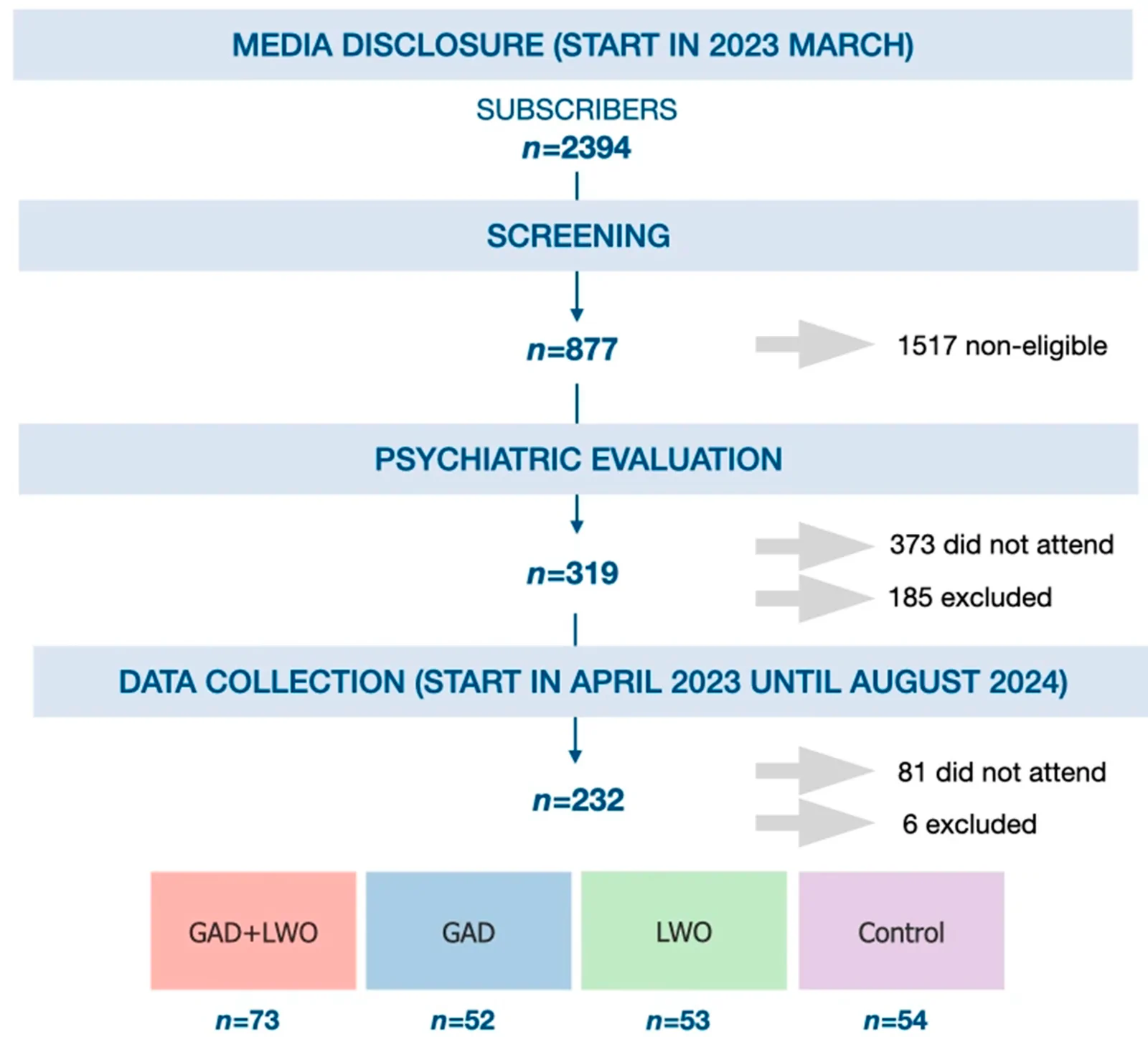
Flow Chart of the study. The diagram illustrates the number of individuals initially contacted, screened, and assessed, as well as the main reasons for exclusion at each stage. The final sample included 232 participants distributed across four groups: Generalized Anxiety Disorder with obesity (GAD + LWO, *n* = 73), Generalized Anxiety Disorder without obesity (GAD, *n* = 52), Living With Obesity without GAD (LWO, *n* = 53), and control participants (*n* = 54).

**Figure 2:**
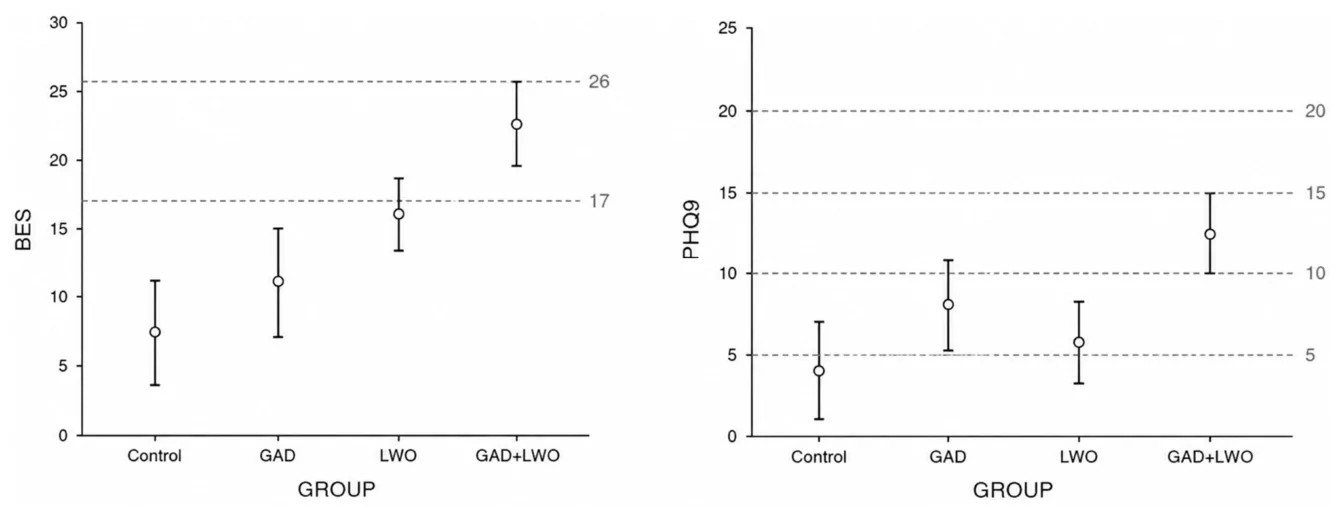
Estimated marginal means of binge eating (BES) and depressive symptoms (PHQ-9) across study groups. Values represent estimated marginal means adjusted for age and sex derived from ANCOVA models. Error bars indicate 95% confidence intervals. Horizontal dashed lines indicate established screening cut-offs for the Binge Eating Scale (BES scores ≤ 17 indicate absence of binge eating, 18–26 suggest moderate binge eating, and ≥27 indicate severe binge eating) and the Patient Health Questionnaire-9 (PHQ-9 levels of depressive symptoms: 0–4 = none or minimal, 5–9 = mild, 10–14 = moderate, 15–19 = moderately severe, and 20–27 = severe depression) and are provided for descriptive reference only.

**Figure 3:**
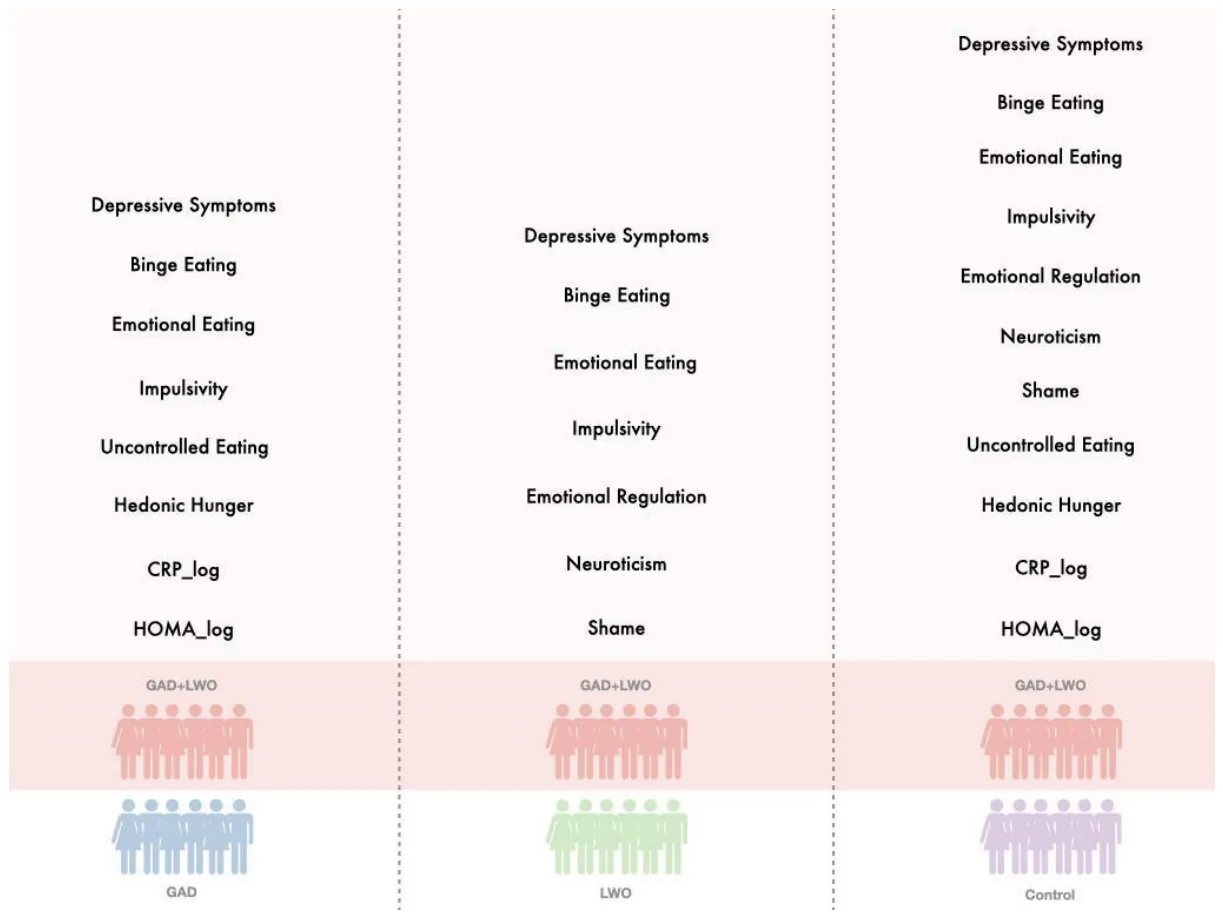
Behavioral, psychological, and biological differences between the GAD + LWO group and the other study groups. Variables shown represent outcomes that differed significantly in post hoc ANCOVA comparisons adjusted for age and sex, with the GAD + LWO group as the reference. Detailed statistical results are presented in Table 3.

**Table 1:** Eligibility criteria for each study group.

Group	Psychiatric Diagnosis	BMI (kg/m^2^)	Key Eligibility Criteria
GAD + LWO	Primary DSM-5 GAD	30.0–39.9	Class I–II obesity No primary MDD; no severe depressive symptoms
GAD	Primary DSM-5 GAD	18.5–24.9	Eutrophic weight No primary MDD; no severe depressive symptoms
LWO	No psychiatric disorder	30.0–39.9	Class I–II obesity -
Controls	No psychiatric disorder	18.5–24.9	Eutrophic weight -

Individuals with overweight (BMI 25.0–29.9 kg/m^2^) and class III obesity (BMI ≥ 40.0 kg/m^2^) were excluded from all groups. All participants underwent the same standardized psychiatric assessment using the M.I.N.I. adapted for DSM-5.

**Table 2:** Demographic characteristics of the sample.

Variable	GAD + LWO (*n* = 73)	LWO (*n* = 53)	GAD (*n* = 52)	Control (*n* = 54)	*p*
Age (years)	36.0 (7.9)	37.6 (7.7)	29.5 (7.2)	29.8 (7.0)	<0.001
Female	61 (83.6%)	36 (67.9%)	48 (92.3%)	49 (90.7%)	0.002
Body fat percentage	44.1 (6.5)	43.1 (7.4)	30.2 (6.6)	29.5 (6.8)	<0.001
Waist circumference	104.8 (9.2)	106.4 (11.2)	74.8 (7.5)	77.8 (7.5)	<0.001
ABEP social class					0.624
A	3 (4.1%)	6 (11.3%)	1 (1.9%)	8 (14.8%)	
B	41 (56.2%)	29 (54.7%)	32 (61.5%)	28 (51.9%)	
C	26 (35.6%)	16 (30.2%)	17 (32.7%)	17 (31.5%)	
D/E	1 (1.4%)	0 (0%)	0 (0%)	0 (0%)	
Missing	2 (2.7%)	2 (3.8%)	2 (3.8%)	1 (1.9%)	
Race/Ethnicity					0.004
White	41 (56.1%)	36 (67.9%)	46 (88.6%)	43 (79.6%)	
Black	14 (19.2%)	9 (17.0%)	2 (3.8%)	6 (11.1%)	
Multiracial	13 (17.8%)	6 (11.3%)	2 (3.8%)	1 (1.9%)	
Others	3 (4.2%)	0 (0%)	0 (0%)	3 (5.5%)	
Missing	2 (2.7%)	2 (3.8%)	2 (3.8%)	1 (1.9%)	

Percentages were calculated using available cases for each variable. The continuous variables are presented as mean (SD) and were compared using one-way analysis of variance (ANOVA). The categorical variables are presented as *n* (%) and were compared using the chi-square test. Abbreviations: Generalized Anxiety Disorder (GAD), Living With Obesity (LWO), Standard Deviation (SD).

**Table 3:** ANCOVA analysis adjusted to age and sex and Post Hoc analysis to characterize behavioral, psychological and biological parameters in patients with GAD + LWO in comparison with other groups (total *N* = 232).

Variables	Group	Estimated Marginal Means (SE)	Cohen’s d (95% CI)	F (df)	η^2^p	*p*	Post Hoc
*BEHAVIORAL*							
Binge Eating Severity (BES) *n* = 230	*GAD + LWO*	*22.1 (0.97)*	*ref.*	38.86 (3, 224)	0.342	0.001 *	1 > 3 * 2 > 3 * 1 > 2
	LWO ^1^	15.0 (1.06) *	0.97 (0.60, 1.35)				
	GAD ^2^	12.0 (1.19) *	1.39 (0.99, 1.79)				
	Control ^3^	7.8 (1.16) *	1.97 (1.55, 2.38)				
Emotional Regulation (DERS) *n* = 225	*GAD + LWO*	*98.9 (2.79)*	*ref.*	13.99 (3, 219)	0.161	0.001 *	2 > 3 * 2 > 1 1 > 3
	LWO ^1^	81.6 (3.04) *	0.84 (0.46, 1.21)				
	GAD ^2^	92.7 (3.41)	0.30 (−0.08, 0.69)				
	Control ^3^	76.9 (3.31) *	1.07 (0.68, 1.46)				
Impulsivity (BIS11) *n* = 225	*GAD + LWO*	*69.6 (1.45)*	*ref.*	15.35 (3, 219)	0.174	0.001 *	2 > 1 2 > 3 1 > 3
	LWO ^1^	59.1 (1.58) *	0.98 (0.60, 1.35)				
	GAD ^2^	62.4 (1.77) *	0.67 (0.28, 1.06)				
	Control ^3^	57.5 (1.72) *	1.13 (0.74, 1.52)				
Emotional Eating (TFEQ) *n* = 225	*GAD + LWO*	*18.6 (0.60)*	*ref.*	15.60 (3, 219)	0.176	0.001 *	1 > 3 * 1 > 2 2 > 3
	LWO ^1^	16.2 (0.64) *	0.55 (0.18, 0.92)				
	GAD ^2^	14.2 (0.72) *	1.02 (0.63, 1.41)				
	Control ^3^	13.4 (0.70) *	1.21 (0.81, 1.60)				
Uncontrolled Eating (TFEQ) *n* = 225	*GAD + LWO*	*24.4 (0.72)*	*ref.*	12.3 (3, 219)	0.145	0.001 *	1 > 3 * 2 > 3 1 > 2
	LWO ^1^	22.0 (0.79)	0.45 (0.08, 0.82)				
	GAD ^2^	19.4 (0.89) *	0.94 (0.54, 1.33)				
	Control ^3^	18.7 (0.86) *	1.06 (0.67, 1.44)				
Cognitive Restraint (TFEQ) *n* = 225	*GAD + LWO*	*13.7 (0.45)*	*ref.*	0.65 (3, 219)	0.009	0.585	
	LWO ^1^	14.5 (0.49)	−0.24 (−0.61, 0.13)				
	GAD ^2^	13.9 (0.55)	−0.06 (−0.45, 0.32)				
	Control ^3^	13.7 (0.53)	0.00 (−0.37, 0.37)				
Hedonic Hunger (PFS) *n* = 225	*GAD + LWO*	*3.2 (0.12)*	*ref*	7.86 (3, 219)	0.097	0.001 *	1 > 2 1 > 3 2 > 3
	LWO ^1^	2.7 (0.13)	0.49 (0.12, 0.86)				
	GAD ^2^	2.5 (0.14) *	0.75 (0.36, 1.14)				
	Control ^3^	2.4 (0.14) *	0.83 (0.45, 1.21)				
Ultra-processed Food Intake (UPF-VIGITEL) *n* = 230	*GAD + LWO*	*3.4 (0.22)*	*ref.*	2.17 (3, 224)	0.028	0.100	
	LWO ^1^	3.0 (0.24)	0.24 (−0.13, 0.60)				
	GAD ^2^	2.8 (0.27)	0.39 (0.01, 0.76)				
	Control ^3^	2.7 (0.27)	0.44 (0.06, 0.82)				
*PSYCHOLOGICAL*							
Anxiety Severity (GAD-7) *n* = 231	*GAD + LWO*	*11.2 (0.52)*	*ref.*	47.32 (3, 225)	0.387	0.001 *	2 > 3 * 2 > 1 * 1 > 3
	LWO ^1^	5.3 (0.57) *	1.51 (1.12, 1.89)				
	GAD ^2^	9.5 (0.65)	0.43 (0.05, 0.81)				
	Control ^3^	3.8 (0.63) *	1.90 (1.49, 2.31)				
Depression Severity (PHQ-9) *n* = 191	*GAD + LWO*	*12.8 (0.65)*	*ref.*	40.32 (3, 185)	0.395	0.001 *	2 > 3 * 2 > 1 1 > 3
	LWO ^1^	5.6 (0.69) *	1.62 (1.19, 2.05)				
	GAD ^2^	8.0 (0.79) *	1.07 (0.65, 1.50)				
	Control ^3^	3.8 (0.79) *	2.04 (1.58, 2.50)				
Neuroticism (ER5FP) *n* = 225	*GAD + LWO*	*6.1 (0.21)*	*ref.*	9.82 (3, 219)	0.119	0.001 *	2 > 3 * 2 > 1 1 > 3
	LWO ^1^	4.9 (0.23) *	0.79 (0.41, 1.17)				
	GAD ^2^	5.8 (0.26)	0.22 (−0.16, 0.61)				
	Control ^3^	4.9 (0.25) *	0.81 (0.43, 1.20)				
Shame (OAS-2) *n* = 225	*GAD + LWO*	*12.4 (0.86)*	*ref.*	10.44 (3, 219)	0.125	0.001 *	2 > 3 * 2 > 1 1 > 3
	LWO ^1^	7.7 (0.95) *	0.73 (0.36, 1.11)				
	GAD ^2^	10.1 (1.06)	0.37 (−0.02, 0.75)				
	Control ^3^	6.3 (1.03) *	0.95 (0.57, 1.34)				
*BIOLOGICAL*							
HOMA-IR-log *[HOMA-IR values]* (HOMA index for insulin resistance) *n* = 217	*GAD + LWO*	*0.32 (0.03 * *[2.4 (0.15)]*	*ref.*	30.47 (3, 211)	0.302	0.001 *	1 > 2 * 1 > 3 * 2 > 3
	LWO ^1^	*0.38 (0.03) * *[2.7 (0.17)]*	−0.28 (−0.65, 0.09)				
	GAD ^2^	0.02 (0.04) * *[1.1 (0.19)]*	*1.32 * *(0.91, 1.73)*				
	Control ^3^	0.02 (0.04) * *[1.2 (0.18)]*	*1.34 * *(0.94, 1.74)*				
CRP-log *[CRP values-mg/L]* (C-Reactive Protein) *n* = 209	*GAD + LWO*	*0.53 (0.05) * *[4.7 (0.53)]*	*ref.*	17.02 (3, 203)	0.201	0.001 *	1 > 2 * 1 > 3 * 2 > 3
	LWO ^1^	0.67 (0.05) *[4.9 (0.59)]*	−0.11 (−0.45, 0.28)				
	GAD ^2^	0.20 (0.06) * [2.1 (0.67)]	0.95 (0.54, 1.36)				
	Control ^3^	0.13 (0.06) * [1.5 (0.64)]	1.16 (0.81, 1.71)				

The total sample size was *N* = 232. The number of participants included in each analysis varied across outcomes due to missing data; the corresponding *n* for each variable is reported in the table. Values are estimated marginal means (SE) adjusted for age and sex. Post hoc pairwise comparisons were conducted using estimated marginal means with the GAD + LWO group, indicated in italics, as the reference. Group numbers used for pairwise comparisons: ^1^, LWO; ^2^, GAD; ^3^, Control. Bonferroni correction was applied for multiple comparisons. Adjusted *p* values were calculated using the Benjamini–Hochberg false discovery rate (FDR) procedure. Bold indicates statistically significant *p*-values (*p* < 0.05). * *p* < 0.05 compared with GAD + LWO. Decimal precision was adapted according to the measurement scale and distribution of variables. Values outside brackets represent the adjusted estimated marginal means obtained from the ANCOVA models using log10-transformed variables. Values in brackets correspond to the observed laboratory measurements in the original units and are provided solely to facilitate clinical interpretation. Abbreviations: GAD, Generalized Anxiety Disorder; LWO, Living With Obesity; SE, standard error.

## Data Availability

The data presented in this study are openly available in Zenodo repository at https://zenodo.org/uploads/18570011 (accessed on 8 September 2026).

## Supplementary Materials

The following supporting information can be downloaded at: https://www.mdpi.com/article/10.3390/nu18193115/s1, Table S1: Sensitivity analyses including main effects of Group, Sex, and Age, as well as the Group × Sex interaction for all behavioral, psychological, and biological outcomes; Table S2: ANCOVA analysis adjusted to age, sex and race/ethnicity and Post Hoc analysis to characterize behavioral, psychological and biological parameters in patients with GAD + LWO in comparison with other groups.
