# Supplementary material for: Behavioral, Psychological, and Metabolic Characteristics of Adults with Co-Occurring Generalized Anxiety Disorder and Obesity: A Cross-Sectional Study
Source: Nutrients. 2026 Sep 22;18(19):3115. doi: 10.3390/nu18193115 (PMC13666706; doi:10.3390/nu18193115)
Supplement: Supplementary file 1 [file nutrients-18-03115-s001.zip › Supplementary Table S1.pdf]

S1 Table - Sensitivity analyses including main effects of Group, Sex, and Age, as well as the Group × Sex interaction for all behavioral, psychological, and biological outcomes.

Outcome	Effect	F	p-value
Binge Eating Severity (BES)	Group	18.366	<.001
Binge Eating Severity (BES)	Sex	10.552	0.001
Binge Eating Severity (BES)	Age	0.811	0.369
Binge Eating Severity (BES)	*Group × Sex*	*0.785*	*0.504*
Emotion Regulation (DERS)	Group	7.817	<.001
Emotion Regulation (DERS)	Sex	4.895	0.028
Emotion Regulation (DERS)	Age	2.440	0.120
Emotion Regulation (DERS)	*Group × Sex*	*0.920*	*0.432*
Impulsivity (BIS-11)	Group	11.388	<.001
Impulsivity (BIS-11)	Sex	0.705	0.402
Impulsivity (BIS-11)	Age	0.217	0.642
Impulsivity (BIS-11)	*Group × Sex*	*1.910*	*0.129*
Emotional Eating (TFEQ-EE)	Group	7.486	<.001
Emotional Eating (TFEQ-EE)	Sex	8.721	0.003
Emotional Eating (TFEQ-EE)	Age	1.881	0.172
Emotional Eating (TFEQ-EE)	*Group × Sex*	*0.436*	*0.728*
Uncontrolled Eating (TFEQ-UE)	Group	4.543	0.004
Uncontrolled Eating (TFEQ-UE)	Sex	2.054	0.153
Uncontrolled Eating (TFEQ-UE)	Age	0.307	0.580
Uncontrolled Eating (TFEQ-UE)	*Group × Sex*	*0.479*	*0.697*
Cognitive Restraint (TFEQ-CR)	Group	0.937	0.424
Cognitive Restraint (TFEQ-CR)	Sex	0.009	0.925

Cognitive Restraint (TFEQ-CR)	Age	0.029	0.864
Cognitive Restraint (TFEQ-CR)	*Group × Sex*	*1.118*	*0.343*
Hedonic Hunger (PFS total)	Group	2.193	0.090
Hedonic Hunger (PFS total)	Sex	5.534	0.020
Hedonic Hunger (PFS total)	Age	0.883	0.348
Hedonic Hunger (PFS total)	*Group × Sex*	*1.450*	*0.229*
Ultra-processed food intake (VIGITEL 24h – UPF)	Group	2.923	0.035
Ultra-processed food intake (VIGITEL 24h – UPF)	Sex	0.019	0.890
Ultra-processed food intake (VIGITEL 24h – UPF)	Age	0.453	0.502
Ultra-processed food intake (VIGITEL 24h – UPF)	*Group × Sex*	*1.418*	*0.238*
Depressive Symptoms (PHQ-9)	Group	19.254	<.001
Depressive Symptoms (PHQ-9)	Sex	0.014	0.905
Depressive Symptoms (PHQ-9)	Age	1.705	0.193
Depressive Symptoms (PHQ-9)	*Group × Sex*	*0.228*	*0.877*
Neuroticism (ER5FP)	Group	7.212	<.001
Neuroticism (ER5FP)	Sex	6.560	0.011
Neuroticism (ER5FP)	Age	0.111	0.740
Neuroticism (ER5FP)	*Group × Sex*	*0.695*	*0.556*
Shame (OAS)	Group	3.266	0.022
Shame (OAS)	Sex	5.837	0.017
Shame (OAS)	Age	2.965	0.086
Shame (OAS)	*Group × Sex*	*0.686*	*0.561*
Insulin resistance HOMA-IR (log)	Group	15.808	<.001

Insulin resistance HOMA-IR (log)	Sex	1.149	0.285
Insulin resistance HOMA-IR (log)	Age	0.288	0.592
Insulin resistance HOMA-IR (log)	*Group × Sex*	*0.544*	*0.653*
C-reactive protein (CRP, log)	Group	7.002	<.001
C-reactive protein (CRP, log)	Sex	4.159	0.043
C-reactive protein (CRP, log)	Age	2.204	0.139
C-reactive protein (CRP, log)	*Group × Sex*	*0.884*	*0.450*

Interaction terms are presented in italics. No Group × Sex interaction was statistically significant for any outcome.
