# Supplementary material for: Behavioral, Psychological, and Metabolic Characteristics of Adults with Co-Occurring Generalized Anxiety Disorder and Obesity: A Cross-Sectional Study
Source: Nutrients. 2026 Sep 22;18(19):3115. doi: 10.3390/nu18193115 (PMC13666706; doi:10.3390/nu18193115)
Supplement: Supplementary file 1 [file nutrients-18-03115-s001.zip › Supplementary Table S2_revised clean.pdf]

S2 Table - ANCOVA analysis adjusted to age, sex and race/ethnicity and Post Hoc analysis to characterize behavioral, psychological and biological parameters in patients with GAD+LWO in comparison with other groups.

Variables	Group	Estimated Marginal Means (SE)	Cohen's d (95% CI)	F (df)	η^2p	P	Post Hoc
BEHAVIORAL							
Binge Eating Severity (BES)	*GAD+LWO*	*22.2 (1.18)*	*ref.*	31.57 (3,214)	0.307	**0.001**	**1>3*** 1>2 **2>3***
	LWO[1]	15.4 (1.36)*	0.94 (0.55, 1.32)				
	GAD[2]	12.5 (1.52)*	1.34 (0.92, 1.77)				
	Control[3]	8.6 (1.39)*	1.88 (1.45, 2.31)				
Emotional Regulation (DERS)	*GAD+LWO*	*98.0 (3.36)*	*ref.*	13.87 (3,216)	0.161	**0.001**	**2>3*** 2>1 1>3
	LWO[1]	80.3 (3.85)*	0.86 (0.48, 1.24)				
	GAD[2]	91.7 (4.31)	0.31 (-0.10, 0.71)				
	Control[3]	75.7 (3.95)*	1.08 (0.67, 1.48)				
Impulsivity (BIS11)	*GAD+LWO*	*70.2 (1.70)*	*ref.*	13.33 (3,216)	0.156	**0.001**	
	LWO[1]	60.1 (1.95)*	0.96 (0.58, 1.34)				
	GAD[2]	64.9 (2.18)	0.51 (0.10, 0.91)				
	Control[3]	59.4 (1.99)*	1.04 (0.63, 1.44)				
Emotional Eating (TFEQ)	*GAD+LWO*	*18.2 (0.71)*	*ref.*	13.59 (3.216)	0.159	**0.001**	**1>3*** 2>3 1>2
	LWO[1]	15.7 (0.81)*	0.57 (0.20, 0.95)				
	GAD[2]	13.8 (0.91)*	1.01 (0.59, 1.42)				
	Control[3]	13.1 (0.83)*	1.19 (0.78, 1.59)				

Uncontrolled Eating (TFEQ)	*GAD+LWO*	*24.4 (0.88)*	*ref.*	9.80 (3,216)	0.120	**0.001**	
	LWO[1]	22.1 (1.00)	0.44 (0.06, 0.81)				
	GAD[2]	19.8 (1.12)*	0.87 (0.46, 1.28)				
	Control[3]	19.0 (1.03)*	1.00 (0.60, 1.40)				
Cognitive Restraint (TFEQ)	*GAD+LWO*	*13.9 (0.54)*	*ref.*	0.798 (3,216)	0.011	0.496	
	LWO[1]	14.6 (0.62)	-0.23 (-0.60, 0.14)				
	GAD[2]	13.8 (0.69)	0.02 (-0.39, 0.42)				
	Control[3]	13.6 (0.63)	0.08 (-0.31, 0.48)				
Hedonic Hunger (PFS)	*GAD+LWO*	*3.1 (0.14)*	*ref*	7.24 (3,216)	0.091	**0.001**	
	LWO[1]	2.6 (0.16)*	0.52 (0.14, 0.89)				
	GAD[2]	2.4 (0.18)*	0.76 (0.35, 1.17)				
	Control[3]	2.4 (0.16)*	0.83 (0.43, 1.23)				
Ultra-processed Food Intake (UPF-VIGITEL)	*GAD+LWO*	*3.4 (0.27)*	*ref.*	2.36 (3,214)	0.032	0.079	
	LWO[1]	2.9 (0.31)	0.30 (-0.08, 0.67)				
	GAD[2]	2.7 (0.35)	0.42 (0.02, 0.82)				
	Control[3]	2.6 (0.32)	0.49 (0.09, 0.88)				
PSYCHOLOGICAL							
Anxiety Severity (GAD-7)	*GAD+LWO*	*11.3 (0.63)*	*ref.*	45.63 (3,215)	0.389	**<0.001**	**2>3*** **2>1*** 1>3
	LWO[1]	5.4 (0.72)*	1.53 (1.13, 1.93)				
	GAD[2]	9.3 (0.81)	0.53 (0.12, 0.93)				
	Control[3]	3.7 (0.74)*	1.96 (1.53, 2.40)				

Depression Severity<br>(PHQ-9)	*GAD+LWO*	*12.4 (0.78)*	*ref.*	36.04<br>(3,175)	0.382	**<0.001**	**2>3***<br>2>1<br>1>3
	LWO[1]	5.1 (0.90)*	1.64<br>(1.19, 2.08)				
	GAD[2]	7.5 (1.02)*	1.11<br>(0.65, 1.57)				
	Control[3]	3.5 (0.94)*	2.00<br>(1.52, 2.49)				
Neuroticism<br>(ER5FP)	*GAD+LWO*	*6.2 (0.25)*	*ref.*	10.13<br>(3,216)	0.123	**0.001**	**2>3***<br>2>1<br>1>3
	LWO[1]	4.9 (0.29)*	0.80<br>(0.42, 1.18)				
	GAD[2]	5.8 (0.33)	0.24<br>(-0.16, 0.64)				
	Control[3]	4.8 (0.30)*	0.86<br>(0.46, 1.26)				
Shame<br>(OAS)	*GAD+LWO*	*13.3 (1.05)*	*ref.*	10.24<br>(3,216)	0.125	**0.001**	**2>3***<br>2>1<br>1>3
	LWO[1]	8.7 (1.20)*	0.71<br>(0.33, 1.09)				
	GAD[2]	11.0 (1.34)	0.35<br>(-0.05, 0.75)				
	Control[3]	7.0 (1.23)*	0.98<br>(0.58, 1.38)				
			BIOLOGICAL				
HOMA-IR_log<br>*[HOMA-IR values]*<br>(HOMA index for insulin resistance)	*GAD+LWO*	*0.28 (0.04)*<br>*[2.21 (0.18)]*	*ref.*	27.77<br>(3,202)	0.292	**0.001**	**1>2***<br>**1>3***<br>3>2
	LWO[1]	*0.34 (0.04)*<br>*[2.52 (0.21)]*	-0.26<br>(-0.64, 0.12)				
	GAD[2]	*-0.03 (0.05)**<br>*[0.94 (0.24)]*	*1.38*<br>*(0.95, 1.81)*				
	Control[3]	*-0.02 (0.04)**<br>*[1.05 (0.22)]*	*1.34*<br>*(0.92, 1.75)*				
CRP_log<br>*[CRP values - mg/L]*<br>(C-Reactive Protein)	*GAD+LWO*	*0.48 (0.06)*<br>*[4.38 (0.69)]*	*ref.*	16.29<br>(3,195)	0.200	**0.001**	**1>2***<br>**1>3***<br>2>3
	LWO[1]	0.52 (0.07)<br>*[4.64 (0.77)]*	-0.12<br>(-0.50, 0.27)				
	GAD[2]	0.13 (0.08)*<br>*[1.73 (0.84)]*	1.03<br>(0.60, 1.46)				
	Control[3]	0.08 (0.07)*<br>*[1.11 (0.78)]*	1.20<br>(0.76, 1.63)				

Values are estimated marginal means (SE) adjusted for age, sex and race/ethnicity. Post hoc pairwise comparisons were conducted using estimated marginal means with the GAD+LWO group as the

reference. Bonferroni correction was applied for multiple comparisons. Adjusted p values were calculated using the Benjamini–Hochberg false discovery rate (FDR) procedure. $^{*}p < 0.05$ compared with GAD+LWO.

Decimal precision was adapted according to the measurement scale and distribution of variables.

Abbreviations: GAD, Generalized Anxiety Disorder; LWO, Living With Obesity; SE, standard error
